# Team running performance while scoring and conceding goals in the UEFA Champions League: analysis of five-minute intervals

**DOI:** 10.1186/s13102-025-01088-4

**Published:** 2025-03-03

**Authors:** Paweł Chmura, Toni Modric, Adrian Drożdżowski, Sime Versic, Damir Sekulic, Marcin Andrzejewski

**Affiliations:** 1 Department of Individual and Team Sports, Wroclaw University of Health and Sport Sciences, St. I.J. Paderewskiego 35, Wrocław, Poland; 2 Faculty of Kinesiology, University of Split, 21000 Split, Croatia; 3 Department of Physiology, Poznań University of Physical Education, Królowej Jadwigi 27/39, 61871 Poznań, Poland

**Keywords:** Soccer, Elite players, Performance analysis, High-intensity running, Match outcome

## Abstract

Performance analysis can provide coaches with a range of relevant information and support more informed decision-making. The objective of this research was to determine running performance (RP) within five-minute intervals when scoring and conceding goals in the UEFA Champions League (UCL). Matches from the UCL 2020/2021 season were analyzed, and relevant data were retrieved using the InStat Fitness semi-automatic video system. Statistical analysis employed one-way analysis of variance (ANOVA) for comparisons and partial eta squared (η2) to determine effect size. Team performance was determined by measuring total distance covered (TD) and high-intensity running (HIR) when the team scored a goal, conceded a goal, and when the score did not change. Our primary results indicated significant differences in three out of 20 five-minute intervals for the TD parameter and four out of 20 for HIR when teams scored goals. There were also significant differences in eight out of 20 intervals for TD and three out of 20 for HIR when teams conceded goals. In conclusion, significant goal concessions were observed during all the five-minute intervals in which teams substantially reduced their RP. From a practical point of view, coaches should be aware, especially in the context of the pacing strategy used, that team RP affects the scoreline directly and the match outcome indirectly.

## Background

In football analysis, it is essential to consider various factors such as collective behavior and technical-tactical performance [1, 2], which play a crucial role in determining match outcome. However, goal-scoring is widely regarded as the ultimate measure of offensive effectiveness and has traditionally been the primary focus of match result analysis [3–6]. As Li et al. [7] points out, goal-scoring patterns can vary and are the result of different styles of play specific to each country, which are shaped by unique cultural, economic and social factors. Unfortunately, much previous research on soccer goals has predominantly focused on developing goal-scoring opportunities or game analysis [4, 6]. As a result, a significant gap exists in the understanding of how the running performance (RP) correlates with the scoreline. This gap provides an opportunity to investigate this relationship by analyzing short time intervals within the match. The validity of such an analysis was demonstrated by, among others, Modric et al. [8] and Kołodziejczyk et al. [9], based on team performance in the UEFA Champions League (UCL). In turn, Wehbe et al. [10] examined the impact of match status and scoreline on team RP in five-minute intervals before and after goals in the Australian Football League. However, there is a lack of information on how teams’ RP relates to scoring or conceding goals within specific five-minute intervals during the match in which they occurred.

The progression of knowledge on physical performance requires exploring the relationship between game time intervals and goal scoring, with the available literature providing many examples of game analyses based on goals scored and conceded. Çebi et al. [11] demonstrated that, during the 2016 European Championships, most goals were scored in the second half (64.8%), with the fewest between one and 15 min (6.5%) and most between 61 and 75 min (29.6%). Similarly, Stafylidis et al. [12] showed that 61.7% of goals were scored in the second half of the game during the 2020–2021 Greek Soccer League season, the fewest goals (9.0%) were scored during the first 15 min, and most (30.6%) were scored during the last 15 min (75—90 +). As shown by Altariba-Bartes et al. [13], when a match is tied and the players are fatigued, a goal scored in the last period of the game can determine victory or defeat. Kołodziejczyk et al. [9] recently attempted to analyze RP over five-minute intervals during the UCL, but not in the context of scoring or conceding goals. Hence, a more detailed analysis, including the shortest possible game intervals, is needed to obtain more accurate data.

Several factors, such as deterioration in the physical and cognitive capabilities of players, increasing fatigue, and tactical decisions of coaches, have been suggested to lead to a higher frequency of goals towards the end of matches. Oliveira and Clemente et al. [14] showed that the total distance covered (TD) by UCL players per game was associated with, among other factors, goals scored and conceded. Moreover, the current data support the fact that team success is related to physical performance, but this conclusion was established for the match outcome rather than match status or fragments of a match [15–17]. Otherwise, Modric et al. [18] stated that winning UCL matches was not strongly influenced by players’ physical performance. Furthermore, teams had similar RP values irrespective of whether they qualified from the group stage into the knockout stage of the UCL, and there were trivial-to-small correlations between RP and total group points [19]. Due to the small number of studies, it is reasonable to conduct more detailed analyses of how parameters such as TD and high-intensity running (HIR) affect the scoreline.

A recent popular line of research involves analyzing the effects of high-intensity activity and its relationship to team success and league position [15, 20, 21]. Such research findings emphasize that coaches should be aware of the importance of building new training strategies to respond to the effects of goals and react with appropriate physical intensity during the match in an attempt to score goals [14]. The relationship between physical performance and goal scoring was also studied in recent years by Klemp et al. [22], who indicated a strong connection between RP and the probability of scoring the first goal. Interestingly, Martínez-Hernández et al. [23] showed that 82.9 ± 1.5% of player involvements included at least one HIR before a goal was scored. Different conclusions were reached by Lago-Peñas et al. [24], who concluded that the players performed less HIR when they were winning than when they were losing during a match. Similar observations showed [18] that a successful match performance (i.e., win) results in lower cumulative HIR. Each of these studies demonstrates that analyzing RP in the context of scoring and conceding goals is a key topic of current and future research on players performing in the UCL.

Based on all of the above findings, the objective of the research was to describe the RP according to goals scored and conceded in the UCL within each five-minute interval. It is expected that the results of this study will allow coaches to, depending on the established pacing strategy, prepare the team for key moments in the game to enhance the efficiency and effectiveness of the team’s performance.

## Methods

### Participants and design

The study investigated elite soccer players from the 2020/21 UCL group stage and obtained data from 20 matches played in groups A (*n* = 3), B (*n* = 3), C (*n* = 4), E (*n* = 4), F (*n* = 3), and G (*n* = 3), to yield 244 observations. Goalkeepers were excluded from the analysis due to the specificity of their position [25]. All data were anonymized in accordance with the principles of the Declaration of Helsinki to ensure player and team confidentiality. The investigation was approved by the local university ethics board (no. 9/2023). As investigation is anonymous and includes adult participants, informed consent was waived. Written permission for data used was obtained from Instat Limited (Limerick, Republic of Ireland, 5 June 2021).

### Data collection and analyses

Data on player RP and goals scored and conceded were collected by the InStat Fitness semi-automatic video system (InStat Limited, Limerick, Republic of Ireland). The system includes three static cameras (i.e., two Full high definition [HD] and one 4 K camera) installed on the roof of the soccer stadium. The system has a sampling frequency of 25 Hz and identifies players by their movement, shape, and color information [26]. The reliability of the InStat system has been validated through comparison with the Vicon system (Vicon Motion Systems, Oxford Metrics, UK) across five velocity bands: 0–7 km/h, 7–15 km/h, 15–20 km/h, 20–25 km/h, and above 25 km/h. This validation process involved analyzing the mean velocity difference (m/s) and mean position difference (m) relative to Vicon. The system has successfully met the official Fédération Internationale de Soccer Association (FIFA) test protocol requirements for Electronic and Performance Tracking Systems (EPTS), with authorization number 1007382 [27]. Previous research further confirms that the system demonstrates high accuracy, along with strong absolute and relative reliability [1, 28, 29].

The RP variables included the TD (m) and HIR (above 19.8 km/h). These metrics have been extensively studied in football analyses due to their established association with team performance in football [16, 30–32].

### Statistical analysis

The Kolmogorov–Smirnov test was used to assess the normality of the analyzed variables, which was accepted for all of them. The data were presented as mean ± standard deviation. (SD). Differences in RP according to the scored/conceded goals in each five-minute period were analyzed by one-way analysis of variance (ANOVA). For this procedure, we introduced dummy variables for each five-minute period, coded as “0” if the team did not score/concede a goal and “1” if the team scored/conceded a goal. These variables were the grouping (independent) variables, while the dependent variables were TD and HIR. The effect size was determined by calculating partial eta squared (η2) (> 0.02 was small, > 0.13 was medium, and > 0.26 was large) [33]. All statistical analyses employed Statistica 13.0 (TIBCO Software Inc., CO, USA).

## Results

### Analysis of goals scored

Table 1 shows the analysis of TD and HIR distance covered in each five-minute interval according to goals scored in the period. For TD, significant differences were observed between 15 and 25 min and after 90 min of play, with goals being associated with lower TD. Between minutes 20 and 25, goals were associated with greater TD covered by teams. In turn, significance was found between minutes 35 and 40 of the first half and in three second-half intervals (50—55, 75—80, and after 90 min) for HIR. Goals were associated with increased running distances between 35 and 40 min and between 50 and 55 min, while significant lower distances were recorded after 75—80 min and after 90 min.

**Table 1:** Differences in total distance covered and high-intensity running (high-speed running and sprinting) in relation to goals scored

Minutes of the match	Goal scored?	Mean ± SD	f-test	p	η2	Mean ± SD	f-test	p	η2
		Total distance				High-intensity running			
0–5	yes	627.80 ± 56.17	0.99	0.32	0.01	44.20 ± 38.07	0.36	0.55	0.01
	no	664.17 ± 81.13				53.67 ± 34.62			
5–10	yes	-	-	-	-	-	-	-	-
	no	619.30 ± 102.53				47.09 ± 30.60			
10–15	yes	596.90 ± 47.00	0.09	0.77	0.01	47.10 ± 24.68	0.43	0.51	0.01
	no	588.42 ± 89.16				55.23 ± 38.85			
15–20	yes	540.35 ± 129.35	5.17	0.02	0.02	38.06 ± 21.54	3.25	0.07	0.01
	no	591.34 ± 114.75				49.05 ± 32.89			
20–25	yes	635.83 ± 78.44	12.48	0.00	0.05	52.21 ± 31.59	3.06	0.08	0.01
	no	555.04 ± 108.9				40.17 ± 32.03			
25–30	yes	552.38 ± 93.96	0.11	0.74	0.01	41.59 ± 28.67	0.93	0.34	0.01
	no	557.78 ± 91.41				46.68 ± 29.64			
30–35	yes	581.68 ± 52.45	0.99	0.32	0.01	43.68 ± 35.01	0.00	0.96	0.01
	no	561.52 ± 87.08				43.36 ± 28.95			
35–40	yes	566.67 ± 84.33	0.97	0.32	0.01	65.87 ± 43.83	6.15	0.01	0.02
	no	585.04 ± 68.83				46.21 ± 28.63			
40–45	yes	549.25 ± 96.79	0.10	0.76	0.01	41.38 ± 26.79	0.00	0.99	0.01
	no	554.93 ± 83.66				41.28 ± 27.19			
45 +	yes	138.20 ± 16.84	0.58	0.45	0.01	10.20 ± 12.68	3.36	0.07	0.01
	no	193.56 ± 161.95				16.43 ± 18.53			
45–50	yes	-	-	-	-	-	-	-	-
	no	633.91 ± 74.55				55.53 ± 32.99			
50–55	yes	594.43 ± 92.86	0.64	0.42	0.01	73.07 ± 36.81	6.36	0.01	0.03
	no	574.76 ± 88.81				50.21 ± 32.71			
55–60	yes	592.25 ± 48.14	0.94	0.33	0.01	40.17 ± 27.78	0.52	0.47	0.01
	no	568.40 ± 84.25				46.69 ± 30.51			
60–65	yes	543.85 ± 57.02	1.20	0.28	0.01	29.85 ± 20.35	1.91	0.17	0.01
	no	515.99 ± 90.69				41.40 ± 29.70			
65–70	yes	533.81 ± 76.76	0.15	0.70	0.01	31.88 ± 26.26	0.65	0.42	0.01
	no	541.15 ± 72.46				37.40 ± 26.55			
70–75	yes	527.50 ± 89.40	0.29	0.59	0.01	39.63 ± 30.70	0.45	0.51	0.01
	no	535.73 ± 77.09				43.23 ± 27.18			
75–80	yes	531.82 ± 61.25	0.00	0.98	0.01	26.91 ± 31.45	9.15	0.00	0.04
	no	532.19 ± 82.33				49.93 ± 34.29			
80–85	yes	485.73 ± 40.43	2.99	0.09	0.01	53.00 ± 29.29	1.57	0.21	0.01
	no	528.35 ± 81.15				41.47 ± 29.84			
85–90	yes	495.00 ± 134.48	3.75	0.05	0.01	33.00 ± 39.18	0.79	0.37	0.01
	no	553.44 ± 91.53				42.32 ± 32.18			
90 +	yes	381.67 ± 57.23	5.97	0.02	0.02	20.28 ± 22.00	4.16	0.04	0.02
	no	444.94 ± 108.5				34.65 ± 29.24			

*SD* Standard deviation, *f-test* Fisher’s exact test, *η2* Eta Squared, *p* Probability

Considering match RP in five-minute intervals, the teams scoring a goal ran the largest TD between minutes 20 and 25, achieving 635.83 ± 78.44 m, and the smallest between 70 and 75 min, obtaining 527.50 ± 89.40 m. Regarding HIR, teams ran the most from 35 to 40 min (65.87 ± 43.83 m) and the least from 75 to 80 min (26.91 ± 31.45 m).

### Analysis of goals conceded

Table 2 shows the analysis of TD and HIR in each five-minute interval according to goals conceded in each period. In the TD analysis, the players obtained the most significant differences up to the 20th minute of the game, between the 25th and 30th minutes, and in the last five minutes of regulation game time of the first half. In addition, significance was recorded in three second-half intervals, between 70 and 75 min and the last 10 min of regular game time. In these intervals, greater TD was associated with fewer conceded goals. Considering HIR, the players obtained the most significant differences between 40 and 45 and 75 and 85 min of play, during which periods increased running distances were observed when teams did not concede goals.

**Table 2:** Differences in total distance covered and high-intensity distance (high-speed running and sprinting) in relation to goals conceded

Minutes of the match	Goal conceded?	Mean ± SD	f-test	p	η2	Mean ± SD	f-test	p	η2
			Total distance				High-intensity running		
0–5	yes	562.80 ± 32.26		0.00	0.03	36.20 ± 22.34	1.27	0.26	0.01
	no	665.53 ± 80.17				53.83 ± 34.79			
5–10	yes	-	-	-		-	-	-	
	no	619.30 ± 102.53				47.09 ± 30.6			
10–15	yes	510.17 ± 42.78	10.51	0.00	0.04	53.00 ± 22.82	0.03	0.86	0.01
	no	592.84 ± 87.65				55.00 ± 39.04			
15–20	yes	495.90 ± 96.59	21.18	0.00	0.08	43.53 ± 24.94	0.57	0.45	0.01
	no	597.34 ± 115.12				48.23 ± 32.71			
20–25	yes	574.57 ± 75.18	0.26	0.61	0.01	44.43 ± 32.86	0.21	0.65	0.01
	no	561.89 ± 111.58				41.07 ± 32.12			
25–30	yes	475.00 ± 100.48	36.18	0.00	0.13	41.21 ± 24.94	1.00	0.32	0.01
	no	570.23 ± 83.05				46.67 ± 30.15			
30–35	yes	530.90 ± 80.57	3.15	0.08	0.01	54.3 ± 35.26	3.03	0.08	0.01
	no	565.97 ± 84.95				42.42 ± 28.69			
35–40	yes	567.50 ± 81.54	1.07	0.30	0.01	59.00 ± 39.51	2.91	0.09	0.01
	no	585.22 ± 68.84				46.50 ± 29.07			
40–45	yes	511.04 ± 107.43	7.46	0.01	0.03	29.84 ± 19.00	5.06	0.03	0.02
	no	559.32 ± 80.70				42.60 ± 27.61			
45 +	yes	76.14 ± 16.54	3.83	0.05	0.01	4.29 ± 5.71	2.98	0.09	0.01
	no	195.86 ± 161.55				16.46 ± 18.61			
45–50	yes	-	-	-		-	-	-	
	no	633.91 ± 74.55				55.53 ± 32.99			
50–55	yes	567.07 ± 85.26	0.15	0.70	0.01	54.36 ± 37.97	0.11	0.74	0.01
	no	576.43 ± 89.34				51.35 ± 33.08			
55–60	yes	597.00 ± 70.37	1.50	0.22	0.01	46.92 ± 27.63	0.00	0.95	0.01
	no	568.03 ± 83.46				46.33 ± 30.57			
60–65	yes	477.33 ± 54.94	3.26	0.07	0.01	32.07 ± 18.08	1.41	0.24	0.01
	no	520.10 ± 90.62				41.35 ± 29.89			
65–70	yes	543.71 ± 57.72	0.03	0.86	0.01	41.06 ± 25.34	0.42	0.52	0.01
	no	540.44 ± 73.70				36.74 ± 26.63			
70–75	yes	503.97 ± 70.77	5.54	0.02	0.02	35.97 ± 22.68	2.18	0.14	0.01
	no	539.19 ± 78.77				43.78 ± 28.15			
75–80	yes	513.81 ± 65.90	1.51	0.22	0.01	34.73 ± 20.71	4.24	0.04	0.02
	no	534.35 ± 81.98				49.42 ± 35.63			
80–85	yes	376.64 ± 61.20	65.55	0.00	0.21	20.07 ± 14.96	8.25	0.00	0.03
	no	535.54 ± 71.83				43.32 ± 30.03			
85–90	yes	497.25 ± 189.53	4.19	0.04	0.02	39.17 ± 33.9	0.09	0.76	0.01
	no	553.83 ± 86.19				42.08 ± 32.45			
90 +	yes	422.95 ± 95.59	0.54	0.46	0.01	39.47 ± 22.88	0.85	0.36	0.01
	no	441.74 ± 107.75				33.10 ± 29.42			

*SD* Standard deviation, *f-test* Fisher’s exact test, *η2* Eta Squared, *p* Probability

Considering match RP in five-minute intervals, the conceding teams ran the greatest TD between 55 and 60 min (597.00 ± 70.37 m) and the least between minutes 25 and 30 (475.00 ± 100.48 m). In addition, for HIR, the teams ran most from 35 to 40 min (59.00 ± 39.51 m) and least from 80 to 85 min (20.07 ± 14.96 m).

## Discussion

The research objective was to assess RP according to goals scored and conceded within five-minute intervals of UCL matches. The analysis was strengthened by its attempt to determine how the short-term RP level of the elite teams affected the scoreline. The main findings were that significant differences were observed in seven out of the 20 five-minute intervals when teams scored and in 11 out of 20 intervals when teams conceded goals. The second main observation was that teams that conceded goals had lower TD and HIR in all indicated intervals, suggesting a potential association between reduced RP and conceding. Thanks to this analysis, coaches can prepare their team and adjust the pacing strategy by optimizing RP to score or avoid conceding at critical moments in the match.

Research on the 2002, 2006, 2010, 2012, and 2016 European Championships has consistently shown that most goals were scored in the final 30 min of the second half [8, 30–32]. Our analysis showed that a change in the scoreline (scoring or conceding a goal) occurred in seven five-minute intervals in the first half and six in the second half when considering TD and HIR. Hence, our results do not support the thesis that it becomes easier to score or concede a goal over time. In turn, Konefał et al. [34] stated that most phases resulting in a match status change occurred during the first half, while all phases affecting the result occurred in the second. These findings may be due to a decline in shooting accuracy caused by player fatigue, which increases as the match progresses and makes scoring more challenging [35]. However, it is worth noting that the difference in running performance for teams that scored goals occurred after 90 min. Another interesting observation is that there is a significant difference when teams also concede goals. Analysis of the last few intervals of the first and second halves of the match showed the highest eta squared value for 80 to 85 min (η2 = 0.21). Its indicating that critical moments that change the scoreline and, consequently, the match outcome, occurred at the end of second halves. This finding implies that the last time intervals of each half of the match demand maximum focus and adjustments in team RP. Moreover, it suggests that coaches should pay attention to supporting and motivating players until the end of both halves, when teams typically lower their RP.

Currently, the level of RP is steadily increasing, and the physical demands for teams are getting higher and higher. As such, a need has arisen to analyze how this affects the scoreline [36]. To minimize the chances of an unfavorable match status, it is important to evaluate RP during short intervals in which teams conceded goals. Our results showed that teams conceding goals had significantly lower TD and HIR in all indicated intervals. Evangelos et al. [4] demonstrated that the first 30 min of the game markedly determined the final match outcome, with studies indicating that the highest number of most demanding passages (MDPs) of play for RP occurs in the first 15 min of the first half [37]. Our results showed that reduced TD in five-minute intervals was associated with an increased probability of conceding a goal (in five intervals of the first half and three of the second half, totaling as much as 40 of the 90 min of play). This was most evident in the first 30 min of the game (η^2^ = 0.08 for the 15–20 min interval and η^2^ = 0.13 for the 25–30 min interval). The relationship between conceding and decreased TD was also highlighted by Konefał et al. [34], who showed that the lowest TD and HIR occurred during the match status changing from winning to drawing, indicating that teams must/should maintain activity throughout the game. Indeed, teams that lowered their TD briefly showed an increased risk of conceding a goal. However, teams with greater TD did not show a higher number of goal-scoring chances (one interval), which may be due to the fact that teams playing at the highest level have more ball possession, better technically advanced players in their lineups, and do not need to cover significantly more TD or HIR than their opponents [8].

HIR is an RP parameter that has recently gained increased attention, with Lago-Penas et al. [38] showing that it increased across all playing positions (ranging from 1.7% to 9.5%) over the last eight La Liga seasons (2012/2013 to 2019/2020). Pranjic et al. [39] suggested that teams achieving greater long-term success at the highest level of football play at a higher game pace. Furthermore, Schulze et al. [40] noted a positive correlation between increased high-intensity physical effort in the minute preceding a goal-scoring opportunity and the match outcome. Our study indicates that HIR analyzed in five-minute intervals less frequently differentiated between teams that scored or conceded goals. Additionally, for teams that scored goals, the HIR results were unclear (significance was shown in two intervals where teams reached higher values and two in which teams decreased HIR). In terms of conceding goals, it is worth highlighting that a similar relationship was noted for TD, with the analysis showing that teams with lower HIR conceded goals in three intervals, two of which occurred in the second half. This suggests that a reduction in HIR during the defending phase, such as fewer accelerations or dynamic changes of direction, is associated with a higher probability of conceding a goal [41]. Coaches should consider these observations and adjust the team’s HIR during play when out of possession, as part of the pacing strategy to manage potential scoreline changes.

In this study, we analyzed selected matches from the UCL group phase, focusing on players who played full matches, disregarding position, match outcome, or contextual factors like playing at home/away or the strength of the opponent. The resulting limitation in the number of observations may have affected the results obtained. Additionally, the lack of RP in four five-minute intervals could have influence the conclusions drawn. Moreover, technical- tactical factors, tactical indicators and club rankings were not analyzed in relation to the differences in RP. Another limitation is that the data were collected during the Covid-19 period, although the influence was equally present for all teams this could have affected the analyzed results due to changes in match conditions, player availability, and potential fatigue factors (the data is representative of the 2020–2021 season, allowing for trend analysis under pandemic conditions). Despite limitations, the study included matches and players participating in the most prestigious soccer competition in the world and provided information on the impact of physical performance on goals scored and conceded. Moreover, the specific timing analysis approach provided insights into short-term RP. Using an analysis based on five-minute intervals should also support coaches’ tactical decisions as matches progress, especially in relation to pacing strategy. Future research could account for the complexity of soccer by incorporating factors that may influence match outcomes and scorelines, such as player positions, technical and tactical indicators. Moreover, it could be expanded to analyze data from multiple seasons, allowing for a more comprehensive understanding of long-term trends, team development, and the influence of evolving strategies over time. As such, continued research into better understanding the impact of RP on the scoreline and match outcome is advised.

## Conclusions

The results of this study confirmed that RP, in terms of TD and HIR, is a valid indicator of goals scored and conceded. In this context, significance was found in seven intervals for scoring and 11 for conceding.

The first observation is that the analysis did not indicate a significant difference in scoring and conceding goals in the first (seven intervals) or second half (six intervals). Nevertheless, RP was relevant in the last intervals of both halves in terms of teams conceding goals. Furthermore, increased RP occurred at 90 + minutes for the team that scored goals.

Our primary results are the greater effect of the TD parameter (11) than the HIR (7) on the teams scoring and conceding goals over five-minute intervals. It was far more evident for conceding (8 to 3). A significant decrease in both parameters resulting in goals conceded. In turn, HIR appeared more crucial in a goal-scoring context (4 to 3), but ambiguous results were obtained for TD.

### Practical applications

When playing soccer, players undertake a variety of decisions and activities that contribute to scoring or conceding goals, including individually and within each formation and team activity of different intensity. Such actions directly impact the scoreline and the match outcome. Our results may be useful for determining RP in the UCL, especially in the context of the pacing strategy used.

The study findings enable the development of team-specific physical training drills that players should undertake more frequently to change an unfavorable match status or maintain a favorable status in appropriate phases [42]. Coaches and analysts should impose physical actions during training sessions, friendly matches, tournament matches, tactical exercises, and game simulations, taking into account the five-minute intervals shown in our research. Training recommendations in soccer should be based on established requirements specific to each game phase, ensuring that players are more equipped to manage their fatigue and, consequently, their tactical responsibilities during the match.

## Data Availability

The data that support the findings of this study are openly available in repository “Zenodo” at University School of Physical Education in Wroclaw. (2024). Team running performance while scoring and conceding goals in the UEFA Champions League: Analysis of five-minute intervals. [Data set]. Zenodo. https://doi.org/10.5281/zenodo.13641908. Futhermore, the datasets used and analyzed during the current study are available from the corresponding author (adrian.drozdzowski@awf.wroc.pl) on reasonable request.
